# Rising Health Care Costs and Life-Cycle Management in the Pharmaceutical Market

**DOI:** 10.1371/journal.pmed.1001461

**Published:** 2013-06-04

**Authors:** Aaron S. Kesselheim

**Affiliations:** Division of Pharmacoepidemiology and Pharmacoeconomics, Department of Medicine, Brigham and Women's Hospital and Harvard Medical School, Boston, Massachusetts, United States of America

## Abstract

Aaron Kesselheim discusses Natalie Vernaz and colleagues' paper on the effects on health care costs of “evergreening” strategies pursued by drug manufacturers.

*Please see later in the article for the Editors' Summary*

**Linked Research Article**

This Perspective discusses the following new study published in *PLOS Medicine*:

Vernaz N, Haller G, Girardin F, Huttner B, Combescure C, et al. (2013) Patented Drug Extension Strategies on Healthcare Spending: A Cost-Evaluation Analysis. PLoS Med 10(6): e1001460. doi:10.1371/journal.pmed.1001460

In a cost-evaluation analysis of pharmacy invoice data in one Canton in Switzerland Nathalie Vernaz and colleagues find that “evergreening” strategies pursued by drug manufacturers have been successful in maintaining market share and contribute to increased overall healthcare costs.

The problem of rising prescription drug costs has emerged as a critical policy issue around the world, particularly in the United States. These costs strain the budgets of patients and health insurers, and directly contribute to adverse health outcomes by reducing adherence to important medications. Drug spending is driven by brand-name drugs, which currently account for about 20% of all prescriptions in the US, but 80% of costs. Brand-name drugs generally are granted periods of market exclusivity during which they charge high prices to account for the initial investment in research and development. In the case of small molecule drugs, once the market exclusivity period ends, competitors producing bioequivalent generic drugs can enter the market, and the drug price quickly falls. In this week's *PLOS Medicine*, Nathalie Vernaz and colleagues study the consequences of this delicate balancing act going awry due to the cumulative effects of so-called life-cycle management (or “evergreening”) strategies employed by the drug's manufacturer [1].

## Life-Cycle Management in the Pharmaceutical Market

The term “life-cycle management” refers to the practice of brand-name manufacturers seeking to further extend the market exclusivity periods for their drugs to maintain revenue streams. Market exclusivity extensions may be achieved through a number of different strategies. Some evergreening strategies offer scant public health benefits, including slight changes in formulation protected by later-issued patents [2], marketing tools such as drug coupons that reduce patients' out-of-pocket spending on brand-name drugs [3], and negotiating settlements with generic companies to prevent challenges to potentially weak or invalid patents [4]. Other evergreening strategies may provide more measurable advantages to patients, such as developing extended-release versions or combination products [5]. Though these latter alterations can enhance convenience and patient adherence, such advantages may also be muted by non-adherence related to the drugs' high costs. In all cases, follow-on products are heavily promoted by the manufacturer to ensure that they are prescribed over the older versions, even if they lack evidence of comparative efficacy or safety [6].

Life-cycle management may contribute to rising costs at a time when government insurance programs are cutting back on important areas of medical coverage, but their impact on costs or health care delivery is not often subject to empirical analysis. In a 2006 publication, my colleagues and I studied three brand-name pharmaceutical products (omeprazole, amoxicillin/clavulanate, and metformin) whose market exclusivities were extended through tactics such as lawsuits against generic competitors and patents on peripheral aspects of products. We identified US$1.5 billion in revenue that Medicaid, the US drug insurance program for low-income patients, could have saved if generic alternatives to these three medications had been available and widely used when the patent on the active ingredient expired [7].

## Excessive Pharmaceutical Spending in the Swiss Canton of Geneva

Research that assesses the implications of life-cycle management in the pharmaceutical market is rare, and the practice continues as health care costs continue to accelerate, which make the analysis by Nathalie Vernaz and colleagues particularly timely. Studying prescription rates in the Swiss canton of Geneva, the authors focused on eight drugs that demonstrate a wide array of evergreening strategies, including single-isomer versions marketed in place of enantiomeric drugs (e.g., levocetirizine and cetirizine), combination products marketed in place of unitary products (e.g., simvastatin/ezetimibe and simvastatin), and slow-release formulations (e.g., extended-release zolpidem and zolpidem). Among all of these examples, the follow-on product had no proven clinical advantage. Vernaz et al. found that the canton, which represents about 5% of Switzerland's total population, would have saved over 30 million Euros between 2001 and 2008 if physicians had avoided the high-cost follow-on products and instead prescribed generic versions of the original products.

In their analysis, Vernaz and colleagues also provide some empirical insight into two possible anti-evergreening tactics. First, they studied the effect on community-based prescribing of a restrictive drug formulary imposed by the region's large academic medical center. They found that requiring prescription of a generic drug (enantiomeric cetirizine) over the follow-on product (single-isomer levocetirizine) in the hospital had “spill-over” effects in encouraging prescribing of the lower-cost product by community physicians, although the policy only saved the health system a small amount. By contrast, listing the follow-on product (single-isomer esomeprazole) over the generic precursor (enantiomeric omeprazole) in the restrictive formulary led to substantial extra costs through the same spill-over mechanism.

Second, their analysis covers a country-wide policy enacted in 2006 to slightly increase out-of-pocket expenses for patients selecting brand-name drugs over generic alternatives. Though they did not specifically test changes in prescription rates before and after this policy change, their results show the limited overall impact that the policy had in stemming prescribing of the second-generation products in their sample.

## Addressing the Public Health Implications of Pharmaceutical Life-Cycle Management

Given the modest effects of restrictive formularies and co-payment changes in combating excessive spending related to drug life-cycle management, what other possible solutions could reduce the negative effects of these tactics on health care systems? Vernaz and colleagues suggest greater investment in comparative efficacy testing of follow-on products to better inform physicians and patients about the benefits—or lack thereof—of higher-cost follow-on products like levocetirizine or simvastatin/ezetimibe. Another more fundamental option would be to treat second-generation products differently under patent statutes or other laws conferring market exclusivity such that less innovative follow-on products earn market exclusivity periods shorter than that of the original products. This pathway was recently upheld in India, where drug patent laws require drugs to demonstrate advances in clinical efficacy, and not merely minor physiochemical differences, to earn market exclusivity [8].

With rigorously collected and analyzed data, the study by Vernaz and colleagues highlights an area of wasteful spending in the health care market. While their manuscript did not directly address patient outcomes, their results suggest that addressing life-cycle management through rational regulatory oversight or alterations in patent or market exclusivity laws will be an important way that policymakers can achieve cost savings without adversely affecting public health.
